# Supplementary material for: High-throughput translational profiling with riboPLATE-seq
Source: Sci Rep. 2022 Apr 5;12:5718. doi: 10.1038/s41598-022-09638-3 (PMC8983706; doi:10.1038/s41598-022-09638-3)
Supplement: Supplementary file 1 — Supplementary Information 1. [file 41598_2022_9638_MOESM1_ESM.pdf]

Supplementary Information

<u>High-Throughput Translational Profiling with riboPLATE-seq</u>

Jordan B. Metz, Nicholas J. Hornstein, Sohani Das Sharma, Jeremy Worley, Christian Gonzalez, Peter A. Sims

Supplementary Figures

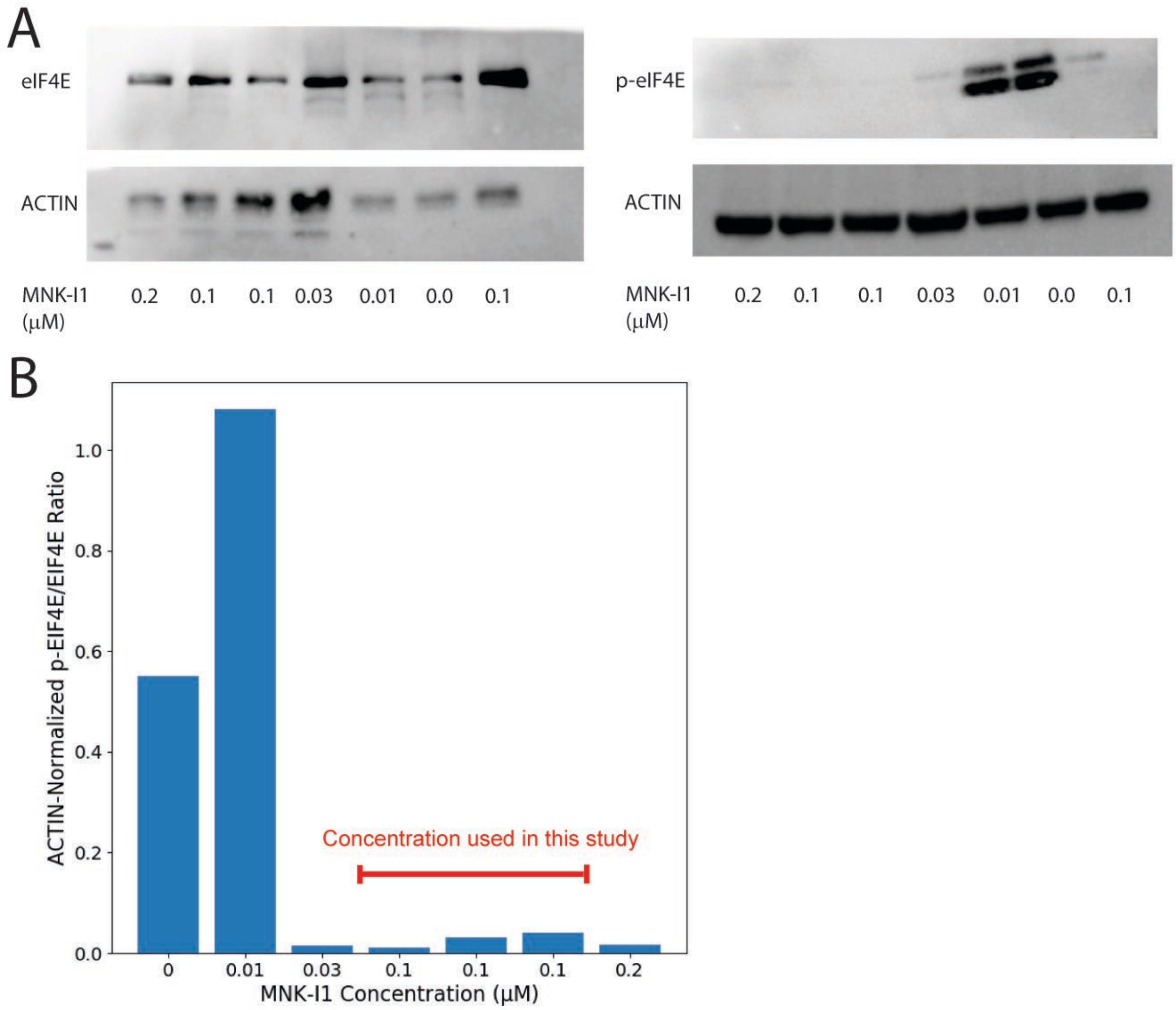

Supplementary Figure 1: Inhibition of eIF4E phosphorylation by MNK-i1 in TS-543 cells. **a)** Western blot of eIF4E (left) and phospho-eIF4E (right) protein levels in comparison to beta-actin under various concentrations of MNK-i1 in TS-543 cells. **b)** Relative quantification ratio of phospho-eIF4E vs total eIF4E, normalized with respect to actin, for each lane in the Western blot. As indicated by the marked depletion of p-eIF4E relative to total eIF4E with the concentration of MNK-i1 used in the study (0.1μM), MNK-i1 blocks eIF4E phosphorylation in TS543 cells at this concentration.

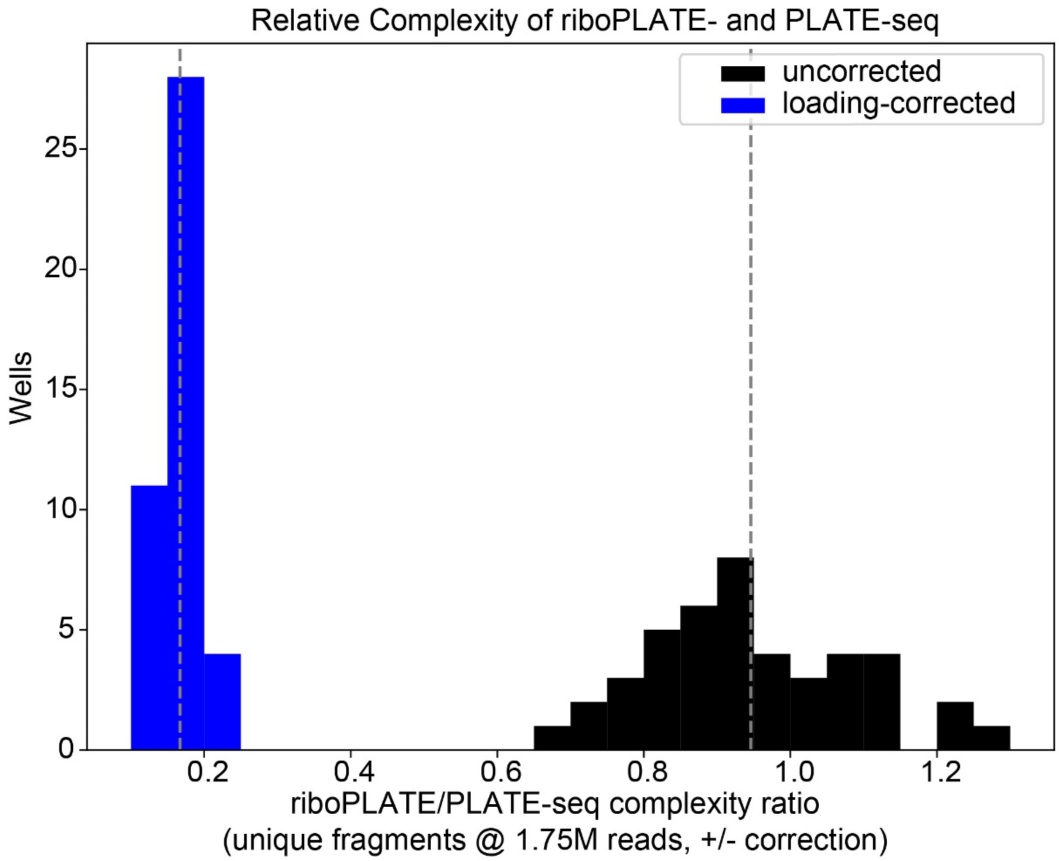

Supplementary Figure 2: Relative complexity of riboPLATE- vs PLATE-seq by uniquely-aligned fragments in TS-543 cells. Histograms of the ratio of riboPLATE- to PLATE-seq complexity matched by sample, across DMSO-treated controls. The black histogram represents the distribution of relative complexity ratios across samples, calculated for each sample as the ratio of uniquely-aligned and distinct fragments contained in 1.75 million randomly-subsampled sequenced reads from its riboPLATE-seq vs PLATE-seq library. This distribution has a mean value near 1, indicating similar complexities of riboPLATE- and PLATE-seq libraries. As 85% of total sample RNA was directed to riboPLATE-seq IP with only the remaining 15% used for PLATE-seq, we corrected this distribution for the difference in input RNA amounts, dividing the ratio for each sample by the RNA loading ratio 85/15. This yielded the corrected distribution (blue histogram) with average corrected complexity ratio 0.17.

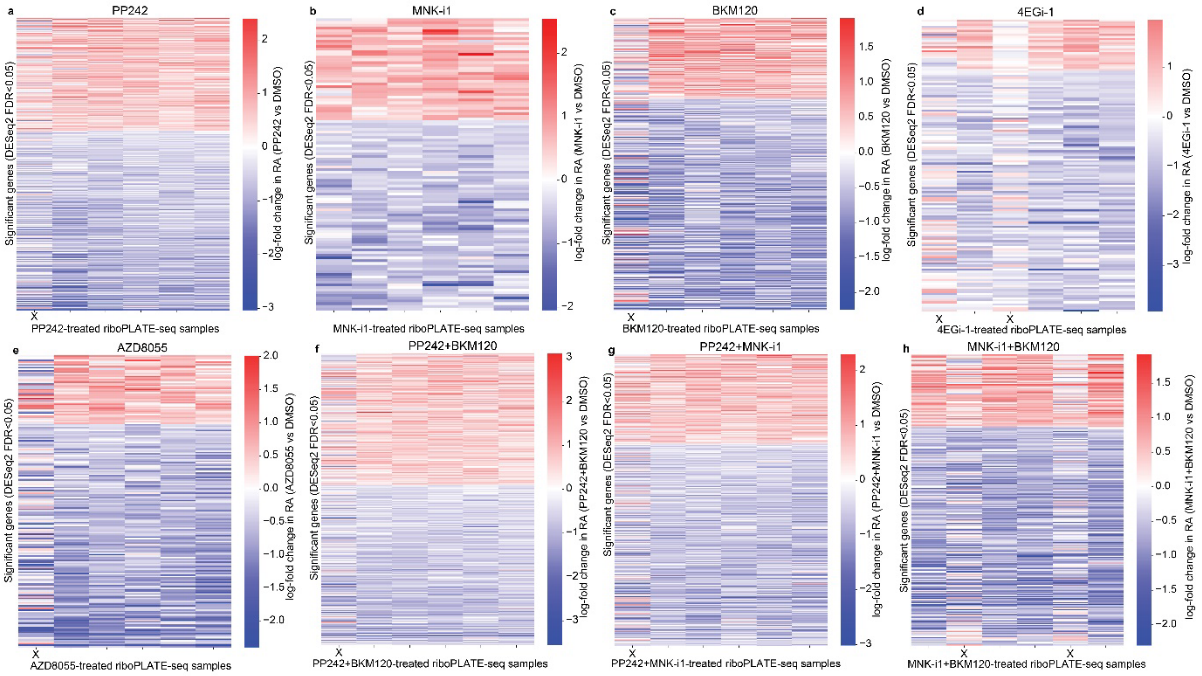

Supplementary Figure 3: Reproducibility of drug-associated log-fold change in RA vs control across riboPLATE-seq replicates for each drug treatment tested in TS-543 cells. Heatmaps of per-sample RA perturbations for **a)** PP242, **b)** MNK-i1, **c)** BKM120, **d)** 4EGi-1, **e)** AZD8055, **f)** PP242+BKM120, **g)** PP242+MNK-i1, and **h)** MNK-i1+BKM120 in comparison to DMSO-treated controls. In each heatmap, the columns represent all samples treated with the drug or combination listed, and the rows contain all genes determined significantly changed in RA (FDR<0.05) by DESeq2 for this treatment. The column for each sample contains its individual log-fold change in RA for all significant genes, relative to the average RA for each gene across DMSO-treated controls, calculated from DESeq2 variance-stabilizing transformation (VST) of riboPLATE- and PLATE-seq counts. Outliers removed from the final dataset are marked with Xs on the X axis.

Supplementary Table Legends

Supplementary Table S1: Per-sample reagent costs. Comparison of per-sample cost for riboPLATE-seq & PLATE-seq vs ligation-free ribosome profiling & RNA sequencing.

Supplementary Table S2: Sequencing library metadata. Includes library construction methods and experimental conditions for each library generated in this study.

Supplementary Table S3: (ribo)PLATE-seq barcodes and sample information. For each of the four PLATE-based libraries generated, a list of barcode sequences for each sample, plus the sample's associated experimental information (drug treatment / ERCC spike-in presence).

Supplementary Table S4: (ribo)PLATE-seq sequencing metrics by sample index. The total number of reads demultiplexed to each sample barcode at various stages of processing and alignment: sequenced, passing adapter-trimming and rRNA depletion pre-processing steps, aligned to the genome, and uniquely aligned to exons of known genes.

Supplementary Data

Supplementary Data S1: Raw scan of the eIF4E (top), p-eIF4E (middle), and Actin (bottom) western blots corresponding to the top left, top right, and bottom left panels of Supplementary Figure 1A, respectively with annotations in red.

Supplementary Data S2: Raw scan of the Actin western blot corresponding to the bottom right panel of Supplementary Figure 1A.

Supplementary Data S3: High-contrast version of Supplementary Data S2 with annotation arrows pointing to the top and bottom boundaries of the blot.
