# Supplementary figures and images for: High-throughput translational profiling with riboPLATE-seq
Source: Sci Rep. 2022 Apr 5;12:5718. doi: 10.1038/s41598-022-09638-3 (PMC8983706; doi:10.1038/s41598-022-09638-3)

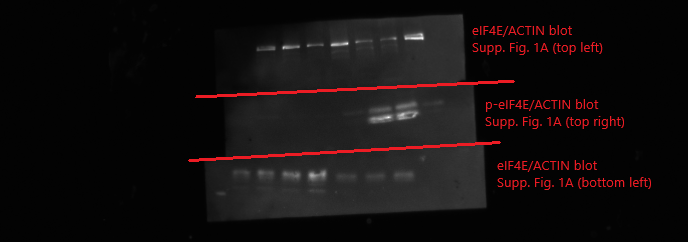

Supplement: Supplementary file 6 — Supplementary Information 6. [file 41598_2022_9638_MOESM6_ESM.tif]

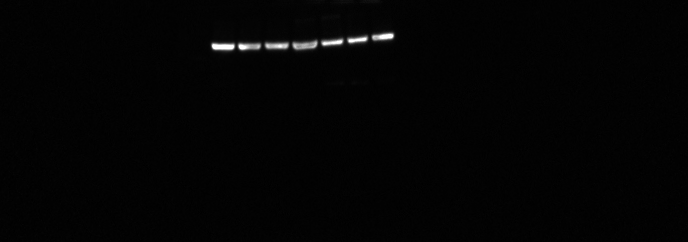

Supplement: Supplementary file 7 — Supplementary Information 7. [file 41598_2022_9638_MOESM7_ESM.tif]

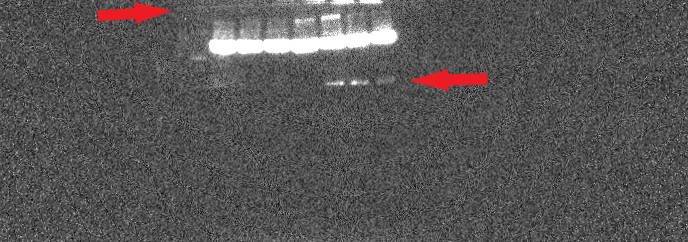

Supplement: Supplementary file 8 — Supplementary Information 8. [file 41598_2022_9638_MOESM8_ESM.tif]
